# Effectiveness of cognitive remediation in schizophrenia: What works and what does not work?

**DOI:** 10.1192/j.eurpsy.2021.148

**Published:** 2021-08-13

**Authors:** A. Vita, G. Deste, A. Ceraso, G. Nibbio, S. Barlati

**Affiliations:** 1 Department Of Clinical And Experimental Sciences University Of Brescia, And Department Of Mental Health And Addiction Services, Spedali Civili Hospital, University of Brescia, Brescia, Italy; 2 Department Of Mental Health And Addiction, ASST Spedali Civili di Brescia, Brescia, Italy; 3 Department Of Clinical And Experimental Sciences, University of Brescia, Brescia, Italy

**Keywords:** schizophrénia, cognition, cognitive remediation

## Abstract

**Introduction:** Cognitive function in schizophrenia is one of the main elements related to functional outcomes. Although there is enough evidence that cognitive remediation (CR) is beneficial, there is still a limited understanding of how the active therapy ingredients contribute to brain changes and translate into improved real-world functioning. **Objectives:** Identify neurobiological, psychopathological, cognitive, and functional CR response or resistance predictors in schizophrenia, considering both cognitive and functional outcomes. **Results:** We still lack a precise understanding of how CR produce its effects with different programs, different numbers of sessions, with and without a therapist. CR exerts its maximal benefit when delivered in the context of psychiatric rehabilitation, but it is not yet clear what are the mechanisms of integrated treatment effectiveness. Only a few studies have looked for the relationship between CR response or resistance and the biological, socio-demographic, clinical and cognitive features in schizophrenia. The current knowledge on efficacy predictors of CR is sparse and include: age, illness duration, premorbid adjustment, baseline cognitive performance, intrinsic motivation, hostility, disorganized symptoms, neurobiological reserve, genetic polymorphisms, antipsychotics amount, the type of CR, etc. All of these limitations may have contributed to the poor implementation of CR in national and international guidelines, as well as in clinical practice. **Conclusions:** It is important that future research shift from studies of group efficacy to individual efficacy of treatments, in the perspective of precision medicine. Issues related to individual effectiveness predictors and interactions between specific pharmacologic, specific CR technique and individual patients’ characteristics should be further addressed.

**Disclosure:**

No significant relationships.

